# Prognostic impact of systolic blood pressure and antithrombotic strategy in patients with atrial fibrillation and stable coronary artery disease: a post-hoc analysis of the AFIRE trial

**DOI:** 10.1038/s41440-025-02449-9

**Published:** 2026-01-05

**Authors:** Shinichi Yamanaka, Takashi Noda, Kotaro Nochioka, Takumi Higuma, Koichi Kaikita, Masaharu Akao, Junya Ako, Tetsuya Matoba, Masato Nakamura, Katsumi Miyauchi, Nobuhisa Hagiwara, Kazuo Kimura, Kunihiko Matsui, Hisao Ogawa, Satoshi Yasuda

**Affiliations:** 1 Department of Cardiovascular Medicine, Tohoku University Graduate, School of Medicine, Sendai, Japan; 2 Cardiovascular Center, Kinki University hospital, Osakasayama, Japan; 3 Department of Cardiology, Kawasaki Municipal Tama Hospital, Kawasaki, Japan; 4 Division of Cardiovascular Medicine and Nephrology, Department of Internal Medicine, Faculty of Medicine, University of Miyazaki, Miyazaki, Japan; 5 Department of Cardiology, National Hospital Organization Kyoto Medical Center, Kyoto, Japan; 6 Department of Cardiovascular Medicine, Kitasato University School of Medicine, Sagamihara, Japan; 7 Department of Cardiovascular Medicine, Faculty of Medical Sciences, Kyushu University, Fukuoka, Japan; 8 Division of Cardiovascular Medicine, Toho University Ohashi Medical Center, Tokyo, Japan; 9 Department of Cardiovascular Medicine Juntendo Tokyo Koto Geriatric Medical Center, Tokyo, Japan; 10 Department of Cardiology, Tokyo Women’s Medical University, Tokyo, Japan; 11 Cardiovascular Center, Yokohama City University Medical Center, Yokohama, Japan; 12 Department of General Medicine and Primary Care, Kumamoto University Hospital, Kumamoto, Japan; 13 Kumamoto University, Kumamoto, Japan

**Keywords:** Atrial fibrillation, Coronary artery disease, Monotherapy, Systolic blood pressure

## Abstract

Backgrounds: The AFIRE (Atrial Fibrillation and Ischemic Events with Rivaroxaban in Patients with Stable Coronary Artery Disease) trial demonstrated that rivaroxaban monotherapy was non-inferior in efficacy and superior in safety compared to rivaroxaban plus single antiplatelet therapy in patients with atrial fibrillation (AF) and stable coronary artery disease (CAD). This study examined whether systolic blood pressure (SBP) affects clinical outcomes and modifies the impact of antithrombotic therapy. Methods: In this post hoc analysis, participants were stratified based on median SBP at baseline: >126 mmHg (High SBP group, *n* = 1042) and ≤126 mmHg (Low SBP group, *n* = 1093). The primary efficacy endpoint was a composite of cardiovascular events and all-cause death. The primary safety endpoint was major bleeding. Results: The mean SBP was 139 mmHg and 114 mmHg in the High and Low SBP groups, respectively. In the propensity score-matched cohort (*n* = 1684), the Low SBP group had a significantly higher incidence of the primary efficacy endpoint (hazard ratio [HR], 1.38; 95% confidence interval [CI], 1.01–1.88; *p* = 0.039), while the primary safety endpoint was comparable between groups. In the Low SBP group, rivaroxaban monotherapy was associated with lower risks of both the primary efficacy (HR, 0.60; 95% CI, 0.41–0.86; *p* = 0.006) and safety endpoints (HR, 0.40; 95% CI, 0.22–0.74; *p* = 0.003) compared with combination therapy, whereas no significant differences were observed in the High SBP group. Conclusions: Lower SBP was associated with increased risk of cardiovascular events and all-cause death. Rivaroxaban monotherapy demonstrated more favorable efficacy and safety outcomes particularly patients with lower SBP.

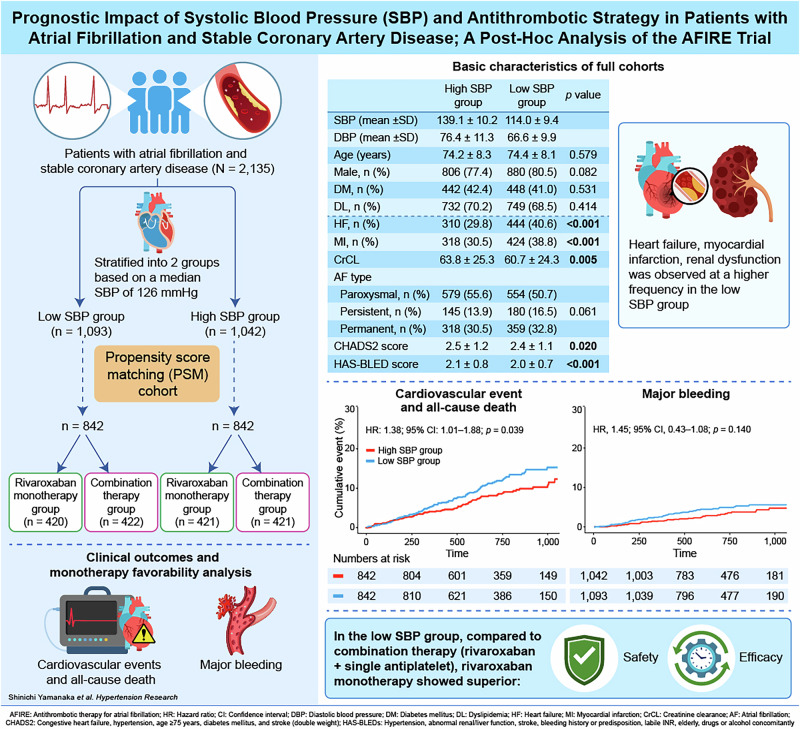

## Introduction

Various risk factors, including atrial fibrillation (AF) and low systolic blood pressure (SBP), contribute to worsening outcomes in patients with stable coronary artery disease (CAD). A systematic review and meta-analysis showed that AF independently increases the risk of death in patients with CAD [1]. Additionally, a composite of cardiovascular death, myocardial infarction, or stroke was higher in patients with stable CAD whose SBP is under 120 mmHg [2]. However, the impact of SBP in patients with both stable CAD and AF remains poorly understood.

Oral anticoagulation is essential for patients with AF due to the risk of thromboembolic events [3–6], while antiplatelet agents are considered the cornerstone of treatment for patients with stable CAD [7–9]. As a result, combination antithrombotic therapy has frequently been used in clinical practice for patients with CAD and AF, increasing the risk of fatal and nonfatal bleeding. The AFIRE trial provided strong evidence that rivaroxaban monotherapy was noninferior to combination therapy with rivaroxaban plus a single antiplatelet agent for major adverse cardiovascular and cerebral events and superior for major bleeding in patients with AF and stable CAD, more than 1 year after revascularization (prior percutaneous coronary intervention [PCI] or coronary artery bypass grafting [CABG]) or in those with angiographically confirmed CAD not requiring revascularization [10]. However, the influence of SBP on the choice of antithrombotic therapy in this population remains unclear.

In this post-hoc analysis of the AFIRE trial, we examined whether SBP influences clinical outcomes and the choice of antithrombotic therapy in patients with AF and stable CAD.

Point of view

- Clinical relevance
  SBP stratification may assist in optimizing antithrombotic therapy among patients with AF and stable CAD, supporting the consideration of rivaroxaban monotherapy.
- Future direction
  Prospective studies involving high risk and other specialized subpopulations are warranted to further assess the effectiveness and safety of oral anticoagulant monotherapy compared with combination therapy.
- Consideration for the Asian population
  Anticoagulant monotherapy may be particularly suitable for high risk Asian populations, and further global investigations are needed to confirm its applicability across diverse clinical settings.

## Methods

### Study population and trial design

The AFIRE trial was a multicenter, randomized, open-label, parallel-group study. The trial design and primary results have been reported previously [11]. In brief, men and women aged ≥20 years with AF and a CHADS₂ score ≥1, as well as stable coronary artery disease CAD, were enrolled. Eligible patients met one of the following criteria: (i) percutaneous coronary intervention (PCI) with or without stenting performed ≥1 year before enrollment; (ii) angiographic or coronary CT evidence of ≥50% coronary stenosis; or (iii) history of coronary artery bypass grafting (CABG) ≥ 1 year before enrollment. Key exclusion criteria included prior stent thrombosis, active malignancy, and poorly controlled hypertension, defined as clinic systolic blood pressure (SBP) ≥ 160 mmHg on two or more occasions. The trial was designed and conducted by an executive steering committee [11].

The study complied with the Declaration of Helsinki and was approved by the institutional review board of the National Cerebral and Cardiovascular Center, Japan, along with the institutional review boards of all participating centers. An independent data and safety monitoring committee was responsible for study monitoring (ID: 20221366). All participants provided written informed consent. A contract research organization (Mebix) assisted with site management, data collection, statistical analysis, and manuscript preparation under the authors’ supervision.

A total of 2240 patients were enrolled, and 2236 were randomly assigned in a 1:1 ratio to receive rivaroxaban monotherapy (10 mg once daily for creatinine clearance 15–49 mL/min, or 15 mg once daily for creatinine clearance ≥50 mL/min) or combination therapy with rivaroxaban plus a single antiplatelet agent (aspirin or a P2Y₁₂ inhibitor), based on the treating physician’s discretion.

In this post-hoc analysis, outcomes were assessed according to systolic blood pressure (SBP) at trial entry in the modified intention-to-treat population. Of the 2215 enrolled patients, 80 lacked SBP data, resulting in 2135 patients available for analysis. Participants were stratified into two groups based on the median baseline SBP: the High SBP group (SBP > 126 mmHg) and the Low SBP group (SBP ≤ 126 mmHg). To address potential confounding by comorbidities, propensity score matching (PSM) was performed. The propensity score was estimated using a logistic regression model including clinically relevant covariates: age, sex, AF type, previous revascularization (PCI or CABG), diabetes mellitus, dyslipidemia, angina, heart failure, myocardial infarction, creatinine clearance, dose of rivaroxaban, and use of proton pump inhibitors. PSM was performed using a greedy nearest-neighbor algorithm without replacement, with a caliper width of 0.05 of the standard deviation of the logit of the propensity score. Balance between groups was assessed using standardized mean differences (SMDs), with values < 0.1 indicating adequate balance. All covariates achieved adequate balance.

### Outcomes

The primary efficacy outcome was a composite of stroke, systemic embolism, myocardial infarction, unstable angina requiring revascularization, and all-cause death. The primary safety outcome was major bleeding, defined according to the criteria of the International Society on Thrombosis and Haemostasis.

### Statistical analyses

Continuous variables are presented as mean ± standard deviation or median with interquartile range, as appropriate. Categorical variables are expressed as counts and percentages. Cumulative event rates were estimated using the Kaplan–Meier method, and incidence rates were reported as percentages per patient-year. Comparisons of outcomes between the High SBP and Low SBP groups, as well as between rivaroxaban monotherapy and combination therapy within each SBP group, were conducted using Cox proportional hazards models. Results are expressed as hazard ratios (HRs) with 95% confidence intervals (CIs).

Randomization in the AFIRE trial was stratified using a minimization method based on age, sex, history of PCI, cardiac insufficiency, heart failure, hypertension, diabetes mellitus, and stroke. All statistical analyses were performed using R (version 4.3.2; R Foundation for Statistical Computing, Vienna, Austria) within RStudio (version 2024.04.2+764 for macOS; Posit Software, PBC, Boston, MA, USA). All reported P-values were two-sided, with values < 0.05 considered statistically significant.

## Results

### Baseline characteristics of the full cohort

**Fig. 1:**
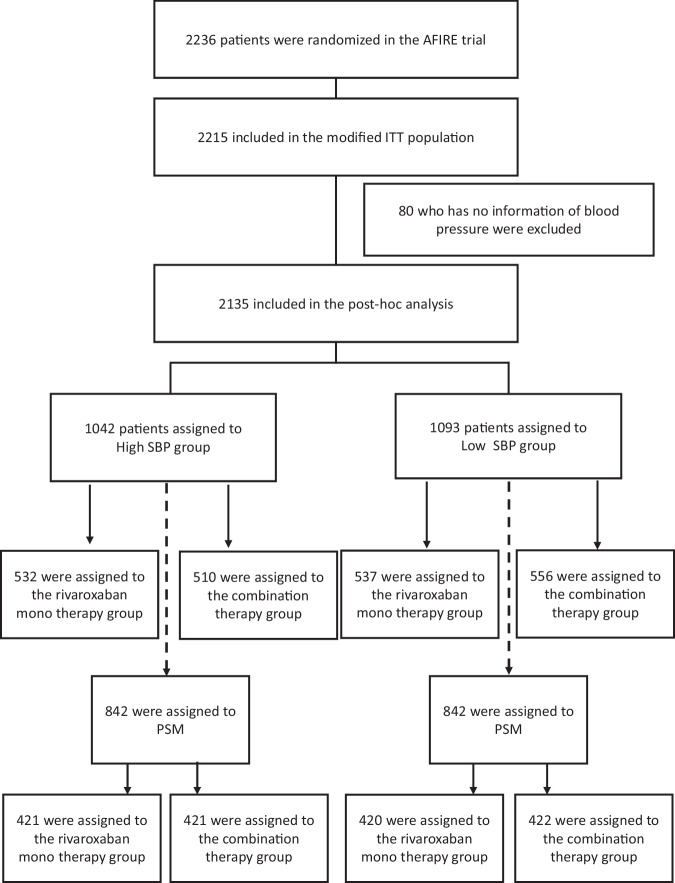
Patient flow of the post-hoc analysis population

Among 2135 patients, 1093 were classified into Low SBP group (SBP ≤ 126 mmHg), while 1042 patients were categorized into High SBP group (SBP > 126 mmHg) (Fig. 1). Table 1 shows the patients’ baseline characteristics. Mean SBP was 114.0 ± 9.4 mmHg in Low SBP group while 139.1 ± 10.2 mmHg in High SBP group. At baseline, the characteristics of two groups were similar, but creatinine clearance and prevalence of prior stroke were significantly lower in Low SBP group, while prior heart failure and myocardial infarction were significantly higher in Low SBP group. Supplementary Fig. 1 shows the relationship between ejection fraction (EF) and SBP in patients with available EF data (*n* = 661). Although the correlation was modest (Pearson’s r = 0.190), it was statistically significant (*P* < 0.001), indicating a positive association between EF and SBP.

**Table 1:** Patients’ clinical characteristics by full cohorts and propensity score-matching cohorts ‘High SBP group’ vs. ‘Low SBP group’

	Full cohorts (*n* = 2135)			PSM cohorts (*n* = 1684)		
	High SBP group (*n* = 1042)	Low SBP group (*n* = 1093)	*P* value	High SBP group (*n* = 842)	Low SBP group (*n* = 842)	*P* value
SBP (mean ± SD)	139.1 ± 10.2	114.0 ± 9.4		138.5 ± 9.8	114.4 ± 9.3	
DBP (mean ± SD)	76.4 ± 11.3	66.6 ± 9.9		76.34 ± 11.4	66.82 ± 9.9	
Age, years	74.2 ± 8.3	74.4 ± 8.1	0.579	74.1 ± 8.3	74.4 ± 8.0	0.454
Male, *n* (%)	806 (77.4)	880 (80.5)	0.082	667(79.2)	663 (78.7)	0.164
AF type						
Paroxysmal, *n* (%)	579 (55.6)	554 (50.7)	0.061	451 (53.6)	438 (52.0)	0.828
Persistent, *n* (%)	145 (13.9)	180 (16.5)		121 (14.1)	129 (15.3)	
Permanent, *n* (%)	318 (30.5)	359 (32.8)		270 (32.1)	275 (32.7)	
Previous PCI, CABG, *n* (%)	797 (76.5)	843 (77.1)	0.765	643 (76.4)	646 (76.7)	0.320
Diabetes mellitus, *n* (%)	442 (42.4)	448 (41.0)	0.531	353 (41.9)	352 (41.8)	0.262
Dyslipidemia, *n* (%)	732 (70.2)	749 (68.5)	0.414	593(70.4)	591 (70.2)	0.481
Angina, *n* (%)	675 (64.8)	679 (62.1)	0.219	521 (61.9)	530 (62.9)	0.004
Heart failure, *n* (%)	310 (29.8)	444 (40.6)	<0.001	285 (33.8)	291 (34.6)	1.000
Stroke, *n* (%)	174 (16.7)	142 (13.0)	0.019	117 (13.9)	121 (14.4)	0.072
Myocardial infarction, *n* (%)	318 (30.5)	424 (38.8)	<0.001	288 (34.2)	289 (34.3)	0.577
Peripheral artery disease, (%)	72(6.91)	60(5.49)	0.203	56(6.65)	49(5.82)	0.545
CrCL	63.8 ± 25.3	60.7 ± 24.3	0.005	63.1 ± 24.5	61.8 ± 25.0	0.262
CHADS2 Score	2.5 ± 1.2	2.4 ± 1.1	0.020	2.5 ± 1.1	2.4 ± 1.1	0.786
CHA2DS2-VASc Score	4.0 ± 1.5	3.9 ± 1.4	0.115	4.0 ± 1.4	4.0 ± 1.4	0.755
HAS-BLED Score	2.1 ± 0.8	2.0 ± 0.7	<0.001	2.1 ± 0.8	2.0 ± 0.7	0.064
Drugs for AF						
Arrythmia drugs	229(21.9)	230(21.0)	0.636	176(20.9)	177(21.0)	1.000
Rate control drugs	391(37.5)	442(49.4)	0.181	328(39.0)	336(39.9)	0.727
None	448(42.9)	452(41.4)	0.469	359(42.6)	356(42.2)	0.921
Dose of rivaroxaban						
10 mg, *n* (%)	466 (44.7)	511 (46.8)	0.720	376 (44.7)	394 (46.8)	0.527
15 mg, *n* (%)	467 (54.4)	570 (52.2)		466 (55.3)	448 (53.2)	
PPI, *n* (%)	618 (59.3)	690(63.1)	0.077	515 (61.2)	517(61.4)	0.229

### Primary efficacy and safety endpoints in the full cohort: comparison between Low SBP and High SBP groups

**Fig. 2:**
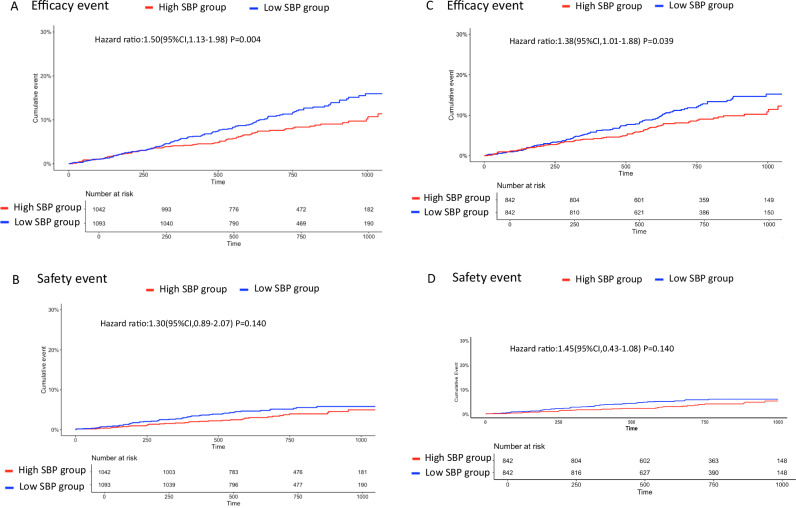
Kaplan–Meier curves for efficacy and safety events in Low SBP group and High SBP Group from the full cohorts and PSM cohorts. Panel **A** shows the efficacy events in Low SBP group and High SBP group from the full cohort. Panel **B** shows the safety events for major bleeding in Low SBP group and High SBP group from the full cohort. Panel **C** shows the efficacy events in Low SBP group and High SBP group from PSM cohorts. Panel **D** shows the safety events for major bleeding in Low SBP group and High SBP group from PSM cohorts

Figure 2A, B shows the primary efficacy and safety endpoints between Low SBP group and High SBP group in full cohorts. Primary efficacy events were significantly higher in Low SBP group than in High SBP group (HR, 1.50; 95% CI, 1.13–1.98; *P* = 0.004). Supplementary Fig. 2 shows the association between systolic blood pressure (SBP) and efficacy outcomes in the overall AFIRE cohort. In the restricted cubic spline analysis (Panel A, knots at the 5th, 35th, 65th, and 95th percentiles; reference SBP = 126 mmHg), the adjusted hazard ratios did not demonstrate statistically significant nonlinearity compared with the linear model (likelihood ratio test, *p* = 0.56). The curve suggested a possible increase in risk at both lower and higher SBP ranges, although the confidence intervals were wide at the distribution tails. Panel B presents the crude distribution of SBP and the observed number of efficacy events in each SBP category. As expected, these crude event rates did not perfectly coincide with the adjusted hazard ratios in Panel A, because the spline model accounts for censoring and confounding, whereas the histogram represents unadjusted data. With respect to primary safety events, there was no statistically difference in Low SBP group compared with High SBP group (HR, 1.30; 95% CI, 0.89–2.07; *P* = 0.14). With respect to the primary efficacy events, the events risk was higher in Low SBP group than in High SBP group across in most of the subgroups. Data are shown in Supplementary Fig. 3.

### Baseline characteristics of the PSM cohort

842 patients were extracted from each group following the PSM procedure (Fig. 1). The baseline characteristics of the PSM cohort are also summarized in Table 1. Mean SBP was 114.4 ± 9.3 mmHg in Low SBP group while 138.5 ± 9.8 mmHg in High SBP group. The baseline covariates were well balanced between the groups, except for a higher prevalence of a history of angina in Low SBP group.

### Primary efficacy and safety endpoints in the PMS cohort; comparison between Low SBP and High SBP groups

Figure 2C, D shows the primary efficacy and safety endpoints between Low SBP group and High SBP group in PSM cohort. The primary efficacy events were significantly higher in Low SBP group than in High SBP group (HR, 1.38; 95% CI, 1.01–1.88; *p* = 0.039) and equivalent safety events (HR, 1.45; 95% CI, 0.43–1.08; *p* = 0.140).

### Clinical significance of monotherapy in Low SBP and High SBP groups

**Fig. 3:**
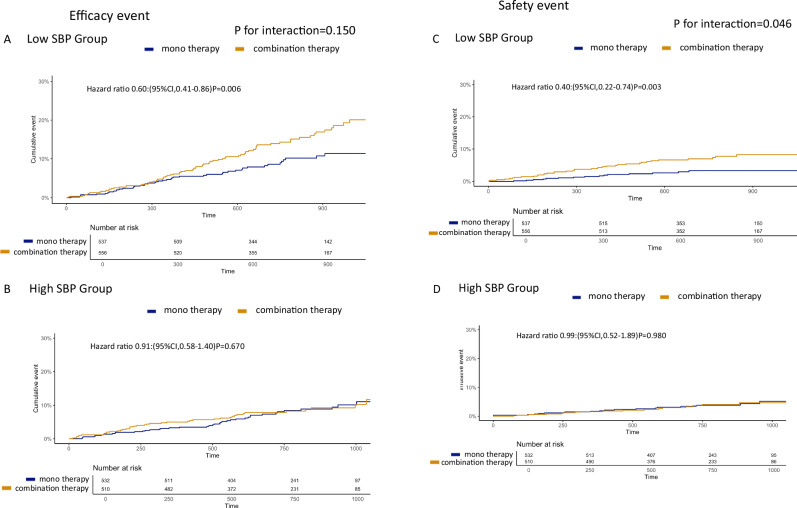
Kaplan–Meier curves for efficacy and safety events in Low SBP group and High SBP between monotherapy and combination therapy. Panel **A** shows efficacy events between monotherapy and combination therapy in Low SBP group. Panel **B** shows efficacy events between monotherapy and combination therapy in High SBP group. Panel **C** shows safety events between monotherapy and combination therapy in Low SBP group. Panel **D** shows safety events between monotherapy and combination therapy in High SBP group

Figure 3A, B shows the difference of the primary efficacy endpoints between monotherapy and combination therapy in Low SBP group and High SBP group. Here, we used the full cohort data. In Low SBP group, monotherapy was superior to combination therapy regarding primary efficacy endpoints (HR, 0.60; 95% CI, 0.41–0.86; *P* = 0.006, Fig. 3A), while there was no statistical difference between monotherapy and combination therapy in High SBP group (Fig. 3B). The interaction between SBP level and treatment effect did not reach statistical significance but showed a trend (*P* for interaction = 0.150).

Regarding the primary safety endpoints, monotherapy was also superior to combination therapy in Low SBP group (HR, 0.40; 95% CI, 0.22–0.74; *P* = 0.003, Fig. 3C). However, in High SBP group, no statistical difference between monotherapy and combination therapy was also observed (HR 0.99; 95% CI, 0.52–1.89; *P* = 0.98, Fig. 3D). The interaction between SBP and treatment was statistically significant, suggesting that the effect of treatment differed by SBP category (*P* for interaction = 0.046).

## Discussion

In this post-hoc analysis of the AFIRE trial, the major findings were as follows: 1) Low SBP was independently associated with a higher risk of the primary efficacy endpoint. 2) Rivaroxaban monotherapy was associated with favorable efficacy and safety outcomes, particularly in patients with low SBP.

### Clinical impact of SBP on primary efficacy endpoints

The optimal SBP range for patients with stable CAD and concomitant AF remains unclear. Previous studies have reported an increased risk of cardiovascular death, myocardial infarction, or stroke in patients with stable CAD when SBP is below 120 mmHg [2]. Study from Taiwan also demonstrated that SBP below 120 mmHg is associated with hospitalization for unstable angina in patients with stable CAD [12]. Additionally, analysis of Rocket AF trial demonstrated that in patients with AF, all-cause mortality is elevated when SBP is below 115 mmHg [13]. Low SBP may reduce perfusion to vital organs, particularly in patients with stable CAD and AF, where compensatory cardiac function is often impaired.

In this post-hoc analysis of the AFIRE trial, the incidence of primary efficacy events was significantly higher in the low SBP group, consistent with previous studies reporting poorer clinical outcomes associated with lower blood pressure [2, 12, 13].

In our cohort, the Low SBP group had a higher prevalence of reduced creatinine clearance, prior myocardial infarction, a history of heart failure, and low EF—factors associated with increased clinical vulnerability. However, although SBP and EF showed a statistically significant correlation (*r* = 0.190), the effect size was weak. This likely reflects the influence of the large sample size, and the clinical significance of this correlation is limited. Additionally, although not statistically significant, nonparoxysmal atrial fibrillation was more common in the Low SBP group, potentially contributing to hemodynamic instability and leading to more frequent use of rate control medications. These characteristics, along with lower SBP, may underlie the higher event rates observed in this group.

To further evaluate the influence of SBP independent of baseline comorbidities, a PSM analysis was performed. Even after adjustment, the low SBP group demonstrated a significantly higher incidence of efficacy endpoints, suggesting that lower SBP itself is associated with worse outcomes in this population. This underscores the need for careful consideration of SBP levels in patients with stable CAD and AF. Maintaining an appropriate SBP range may help support adequate organ perfusion and improve overall prognosis in this complex clinical setting.

Data from the prospective observational longitudinal registry of patients with stable coronary artery disease (CLARIFY) registry demonstrated that not only low but also elevated SBP levels are associated with increased cardiovascular risk in patients with stable CAD [2]. For instance, compared with a reference SBP group of 120–129 mmHg, the HR for the primary outcome was 1.51 (95% CI, 1.32–1.73) in patients with SBP of 140–149 mmHg, and 2.48 (95% CI, 2.14–2.87) in those with SBP ≥ 150 mmHg. In addition, Systolic Blood Pressure Intervention Trial (SPRINT) demonstrated that intensive SBP lowering to <120 mmHg, compared with a target of <140 mmHg, reduced fatal and nonfatal cardiovascular events and all-cause mortality among high-risk individuals without diabetes [14]. Similar benefits of intensive blood pressure control have been reported in patients with type 2 diabetes as well [15].

The present post-hoc analysis of the AFIRE trial showed a U-shaped association between SBP and the risk of primary efficacy event as similar to the previous analysis such as that of CLARIFY registry. Moreover, within the component of primary efficacy events, the all cause death was significantly increased in Low SBP group compared to High SBP group in full cohorts (HR, 1.61; 95% CI, 1.10–2.36; *p* = 0.014), while it showed a same tendency in PSM cohorts (HR, 1.44; 95% CI, 0.94–2.20; *p* = 0.095). Data of components are shown in Supplementary Tables 1, 2. This result is consistent with the analysis of the ROCKET-AF [13], demonstrating the poor mortality in lower SBP population in AF patients. In addition, in the present analysis, the component of unstable angina pectoris also demonstrated higher prevalence in Low SBP group than in High SBP group in both Full cohorts (HR, 3.19; 95% CI, 1.37–7.44; *p* = 0.007) and PSM cohorts (HR, 2.73; 95% CI, 1.07–6.98; *p* = 0.035). This is also consistent with the previous study [12], demonstrating the highly cardiac events in lower SBP population in stable CAD population.

Our findings were similar to a combination of the results reported separately in patients with AF and stable CAD.

### Clinical impact of SBP on primary safety endpoints

Regarding primary safety events, the HAS-BLED score was significantly lower in the Low SBP group compared with the High SBP group (Low SBP group: 2.0 ± 0.7, High SBP group: 2.2 ± 0.8, *P* < 0.001). However, there was no significant difference in the primary safety endpoints between the Low SBP and High SBP groups. In another post-hoc analysis of the AFIRE trial, no difference in HAS-BLED scores was observed between patients with bleeding events and those without bleeding events [16]. While the HAS-BLED score was designed to assess bleeding risk in patients on warfarin, it did not effectively differentiate bleeding risk in the AFIRE trial, despite a higher prevalence of previous myocardial infarction and/or heart failure in the Low SBP group, conditions that could increase the risk of bleeding [17, 18].

### Clinical significance of monotherapy in Low SBP group

As bleeding is closely associated with subsequent cardiovascular events in patients with CAD [19], minimizing bleeding risk is essential in the context of antithrombotic therapy. A recent sub-analysis of the AFIRE trial indicated that patients who experienced major bleeding had a higher incidence of cardiovascular events, and that major bleeding was associated with increased morbidity and mortality [16]. In addition, with regarding to the primary safety events, patients who had revascularization of multi vessel or left main trunk had a superior efficacy of mono therapy compared with mono vessel [20]. Previous analysis of the AFIRE trial demonstrated that in patients with a history of revascularization and heart failure, mono therapy resulted in more favorable safety and efficacy compared to combination therapy respectively [21, 22]. These results suggest that monotherapy plays a superior role in terms of reducing both ischemic and bleeding events especially in patients with poor clinical background. In the present analysis, the Low SBP group included more patients with a history of myocardial infarction, renal dysfunction and/or heart failure, factors that may contribute to an elevated ischemic and bleeding risk. In this higher-risk population, rivaroxaban monotherapy may offer a clinical advantage by reducing both efficacy and safety events compared with combination therapy, which is consistent with previous studies involving multiple comorbidities. The original AFIRE trial demonstrated that rivaroxaban monotherapy was noninferior to combination therapy regarding efficacy, and superior with respect to safety, in patients with AF and stable CAD [10]. In the current post-hoc analysis, the benefit of monotherapy appeared to be particularly pronounced in the Low SBP group, which included patients with poor clinical backgrounds. These findings support the use of rivaroxaban monotherapy in this subgroup as a reasonable strategy to balance ischemic and bleeding risks.

### Limitations

This study has several limitations. First, although the overall sample size was substantial, subgroup analyses reduced the number of patients in each category, potentially limiting statistical power. Second, SBP was assessed only at baseline; therefore, changes during follow-up were not captured, making it difficult to evaluate the longitudinal impact of blood pressure. Third, patients with uncontrolled hypertension—defined as clinic SBP ≥ 160 mmHg on two or more occasions—were excluded from the trial, which may have reduced the incidence of adverse events, particularly in the High SBP group, and influenced comparative outcomes. Fourth, the Low SBP group likely included a heterogeneous population, encompassing both patients with well-controlled blood pressure and those with underlying conditions contributing to low SBP, which may have affected results. Finally, detailed data on medications such as angiotensin II receptor blockers and mineralocorticoid receptor antagonists were not available, limiting the ability to assess their potential influence on outcomes.

### Perspective of Asia

Asian populations are characterized by a higher susceptibility to bleeding events [23, 24]. The AFIRE trial, which demonstrated the clinical utility of direct oral anticoagulant monotherapy in patients with AF and CAD, provided robust evidence derived from an Asian cohort [10]. This treatment concept has subsequently been validated in Western populations through the AQUATIC trial [25]. The present sub analysis of the AFIRE study further extends this evidence by identifying SBP as a prognostic factor influencing clinical outcomes in patients with AF and CAD.

## Conclusion

This post-hoc analysis of the AFIRE trial demonstrated that low systolic blood pressure was independently associated with a higher risk of efficacy events in patients with AF and stable CAD. In this high-risk population, rivaroxaban monotherapy was associated with more favorable safety and efficacy outcomes than combination therapy, supporting its use in clinical practice.

## Supplementary information

Supplementary Figure and tables legends

Supplementary Tables

Supplementary Figures

Supplementary File

The list of AFIRE investigators
