# Supplementary material for: Prognostic impact of systolic blood pressure and antithrombotic strategy in patients with atrial fibrillation and stable coronary artery disease: a post-hoc analysis of the AFIRE trial
Source: Hypertens Res. 2026 Jan 5;49(4):1139–49. doi: 10.1038/s41440-025-02449-9 (PMC13050638; doi:10.1038/s41440-025-02449-9)
Supplement: Supplementary file 1 — Supplementary Figure and tables legends [file 41440_2025_2449_MOESM1_ESM.docx]

**Supplementary Figure 1 shows the relationship between SBP and available EF.**

**Supplementary Figure 2 shows the association between SBP and risk of primary efficacy events in the full cohort.** Panel A shows the Cox proportional hazards regression with restricted cubic splines. Panel B shows the crude distribution of SBP and observed number of efficacy events in each SBP category.

**Supplementary Figure 3 shows the subgroup analysis of efficacy events in Low SBP group and High SBP group in full cohort.**

**Supplementary Table 1 shows the individual components of efficacy and safety events between Low SBP and High SBP group of the Full cohorts.**

**Supplementary Table 2 shows the individual components of efficacy and safety events between Low SBP and High SBP group in the PSM cohorts.**
