# Supplementary material for: Prognostic impact of systolic blood pressure and antithrombotic strategy in patients with atrial fibrillation and stable coronary artery disease: a post-hoc analysis of the AFIRE trial
Source: Hypertens Res. 2026 Jan 5;49(4):1139–49. doi: 10.1038/s41440-025-02449-9 (PMC13050638; doi:10.1038/s41440-025-02449-9)
Supplement: Supplementary file 2 — Supplementary Tables [file 41440_2025_2449_MOESM2_ESM.docx]

**Supplementary Table 1:**

**Individual components of Efficacy and Safety events between Low SBP and High SBP group of the Full cohorts.**

| Endpoint | Low SBP  N=1093 | | High SBP  N=1042 | Hazard ratio  (95% CI) | P value |
| --- | --- | --- | --- | --- | --- |
| Primary Efficacy Endpoint | | 125(11.44%) | 81(7.77%) | 1.50  (1.13-1.98) | 0.004 |
| Cerebral apoplexy | 7(0.64%) | | 10(0.96%) | 1.48  (0.56-3.89) | 0.424 |
| Unstable angina pectoris | 23(2.1%) | | 7(0.67%) | 3.19  (1.37-7.44) | 0.007 |
| Myocardial infarction | 10(0.91%) | | 10(0.96%) | 1.04  (0.43-2.50) | 0.926 |
| Cerebral infarction | 29(2.65%) | | 20(1.92%) | 1.39  (0.79-2.46) | 0.250 |
| Embolism | 0(0) | | 3(0.29%) | - | - |
| Death | 70(6.4%) | | 42(4.03%) | 1.61  (1.10-2.36) | 0.014 |
| Primary Safety  Endpoint | 52(4.76%) | | 37(3.55%) | 1.30  (0.89-2.07) | 0.140 |

**Supplementary Table 2:**

**Individual components of Efficacy and Safety events between Low SBP and High SBP group in the PSM cohorts**

| Endpoint | Low SBP  N=842 | High SBP  N= 842 | Hazard ratio  (95% CI) | P value |
| --- | --- | --- | --- | --- |
| Primary Efficacy Endpoint | 95(11.28%) | 70(8.31%) | 1.38  (1.01-1.88) | 0.039 |
| Cerebral Apoplexy | 7(0.83%) | 8(0.95%) | 0.89  (0.32-2.64) | 0.824 |
| Unstable angina pectoris | 16(%) | 6(%) | 2.73  (1.07-6.98) | 0.035 |
| Myocardial infarction | 8(0.95%) | 9(1.06%) | 0.90  (0.35-2.32) | 0.821 |
| Cerebral infarction | 23(2.73%) | 17(2.01%) | 1.37  (0.73-2.57) | 0.321 |
| Embolism | 0(0) | 2(0) | - | - |
| Death | 51(6.05%) | 36(4.27%) | 1.44  (0.94-2.20) | 0.095 |
| Primary Safety  Endpoint | 44(5.22%) | 32(3.80%) | 1.45  (0.43-1.08) | 0.140 |
