# Supplementary material for: Prognostic impact of systolic blood pressure and antithrombotic strategy in patients with atrial fibrillation and stable coronary artery disease: a post-hoc analysis of the AFIRE trial
Source: Hypertens Res. 2026 Jan 5;49(4):1139–49. doi: 10.1038/s41440-025-02449-9 (PMC13050638; doi:10.1038/s41440-025-02449-9)
Supplement: Supplementary file 3 — Supplementary Figures [file 41440_2025_2449_MOESM3_ESM.pptx]

## Slide 1
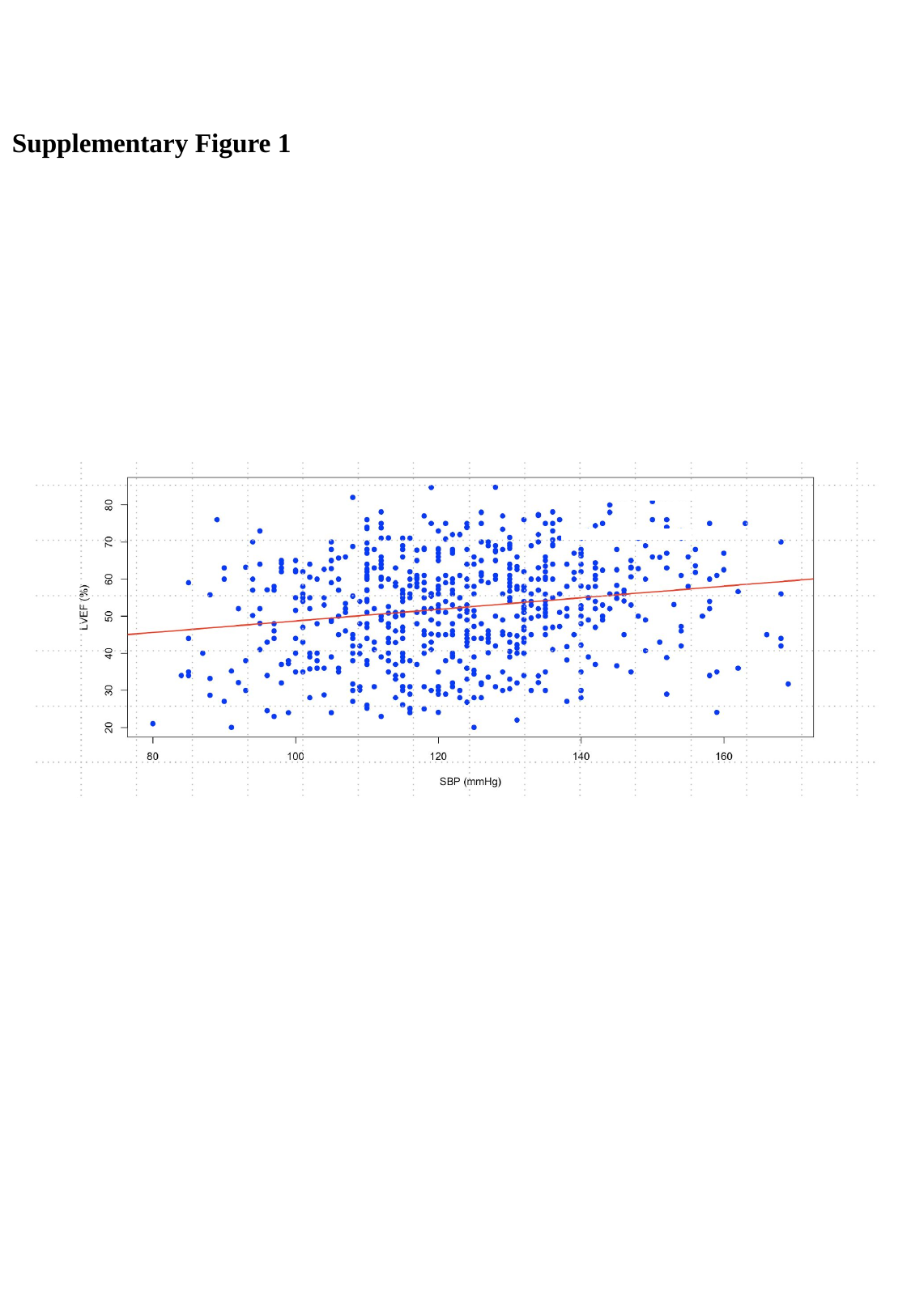

Supplementary Figure 1

## Slide 2
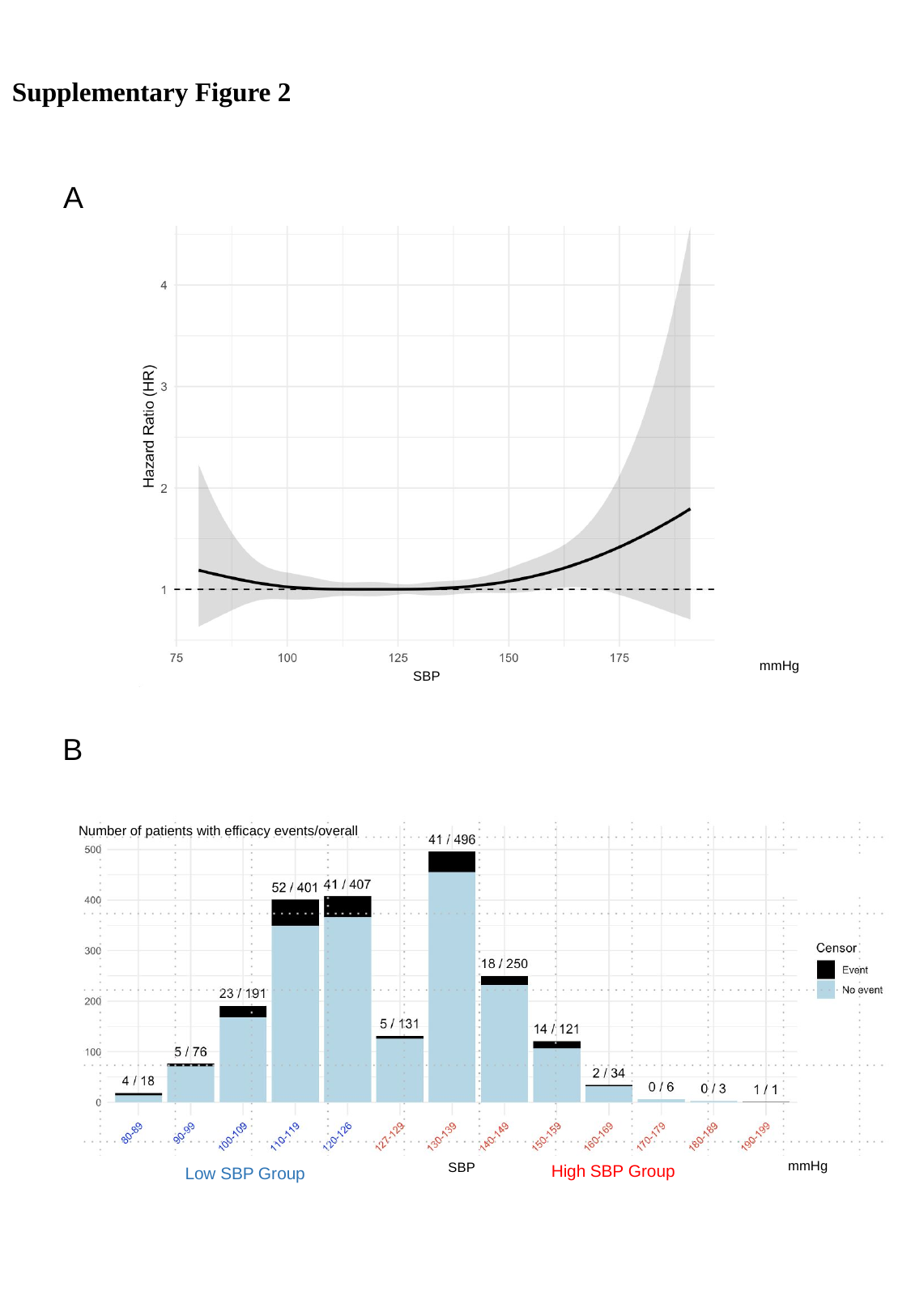

Supplementary Figure 2
A
mmHg
SBP
B
Number of patients with efficacy events/overall
mmHg
SBP
High SBP Group
Low SBP Group

## Slide 3
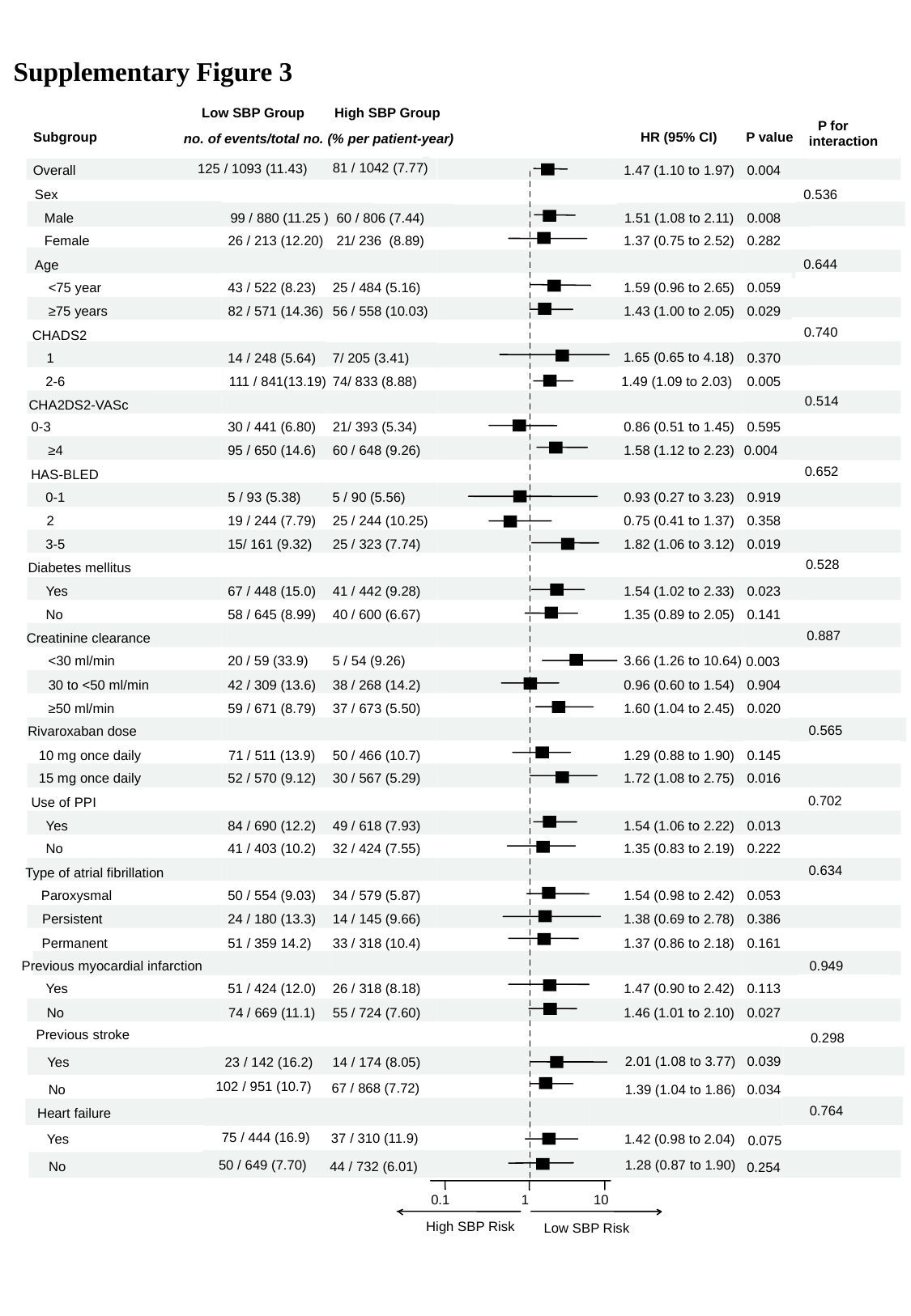

Supplementary Figure 3
Low SBP Group
High SBP Group
P for
Subgroup
HR (95% CI)
P value
 interaction
81 / 1042 (7.77)
125 / 1093 (11.43)
Overall
1.47 (1.10 to 1.97)
0.004
0.536
Sex
 Male
99 / 880 (11.25 )
 60 / 806 (7.44)
1.51 (1.08 to 2.11)
0.008
 Female
26 / 213 (12.20)
 21/ 236 (8.89)
1.37 (0.75 to 2.52)
0.282
0.644
Age
 <75 year
43 / 522 (8.23)
25 / 484 (5.16)
1.59 (0.96 to 2.65)
0.059
 ≥75 years
82 / 571 (14.36)
56 / 558 (10.03)
1.43 (1.00 to 2.05)
0.029
0.740
CHADS2
1.65 (0.65 to 4.18)
　　　　　2
 1
14 / 248 (5.64)
7/ 205 (3.41)
0.370
1.49 (1.09 to 2.03)
 2-6
111 / 841(13.19)
74/ 833 (8.88)
0.005
0.514
CHA2DS2-VASc
 0-3
30 / 441 (6.80)
21/ 393 (5.34)
0.86 (0.51 to 1.45)
0.595
 ≥4
95 / 650 (14.6)
60 / 648 (9.26)
1.58 (1.12 to 2.23)
0.004
0.652
HAS-BLED
 0-1
5 / 93 (5.38)
5 / 90 (5.56)
0.93 (0.27 to 3.23)
0.919
 2
19 / 244 (7.79)
25 / 244 (10.25)
0.75 (0.41 to 1.37)
0.358
 3-5
15/ 161 (9.32)
25 / 323 (7.74)
1.82 (1.06 to 3.12)
0.019
0.528
Diabetes mellitus
 Yes
67 / 448 (15.0)
41 / 442 (9.28)
1.54 (1.02 to 2.33)
0.023
 No
58 / 645 (8.99)
40 / 600 (6.67)
1.35 (0.89 to 2.05)
0.141
0.887
Creatinine clearance
0.003
 <30 ml/min
20 / 59 (33.9)
5 / 54 (9.26)
3.66 (1.26 to 10.64)
 30 to <50 ml/min
42 / 309 (13.6)
38 / 268 (14.2)
0.96 (0.60 to 1.54)
0.904
 ≥50 ml/min
59 / 671 (8.79)
37 / 673 (5.50)
1.60 (1.04 to 2.45)
0.020
0.565
Rivaroxaban dose
 10 mg once daily
71 / 511 (13.9)
50 / 466 (10.7)
1.29 (0.88 to 1.90)
0.145
 15 mg once daily
52 / 570 (9.12)
30 / 567 (5.29)
1.72 (1.08 to 2.75)
0.016
0.702
Use of PPI
 Yes
84 / 690 (12.2)
49 / 618 (7.93)
1.54 (1.06 to 2.22)
0.013
 No
41 / 403 (10.2)
32 / 424 (7.55)
1.35 (0.83 to 2.19)
0.222
0.634
Type of atrial fibrillation
 Paroxysmal
50 / 554 (9.03)
34 / 579 (5.87)
1.54 (0.98 to 2.42)
0.053
 Persistent
24 / 180 (13.3)
14 / 145 (9.66)
1.38 (0.69 to 2.78)
0.386
 Permanent
51 / 359 14.2)
33 / 318 (10.4)
1.37 (0.86 to 2.18)
0.161
0.949
Previous myocardial infarction
 Yes
51 / 424 (12.0)
26 / 318 (8.18)
1.47 (0.90 to 2.42)
0.113
 No
74 / 669 (11.1)
55 / 724 (7.60)
1.46 (1.01 to 2.10)
0.027
0.1
1
10
High SBP Risk
no. of events/total no. (% per patient-year)
Previous stroke
 0.298
 Yes
2.01 (1.08 to 3.77)
0.039
14 / 174 (8.05)
23 / 142 (16.2)
102 / 951 (10.7)
67 / 868 (7.72)
0.034
No
1.39 (1.04 to 1.86)
 0.764
Heart failure
0.075
75 / 444 (16.9)
37 / 310 (11.9)
Yes
1.42 (0.98 to 2.04)
 No
50 / 649 (7.70)
1.28 (0.87 to 1.90)
44 / 732 (6.01)
0.254
Low SBP Risk
