# Supplementary material for: Prognostic impact of systolic blood pressure and antithrombotic strategy in patients with atrial fibrillation and stable coronary artery disease: a post-hoc analysis of the AFIRE trial
Source: Hypertens Res. 2026 Jan 5;49(4):1139–49. doi: 10.1038/s41440-025-02449-9 (PMC13050638; doi:10.1038/s41440-025-02449-9)
Supplement: Supplementary file 4 — Supplementary File [file 41440_2025_2449_MOESM4_ESM.docx]

Supplementary File contains Supplementary Figure 1-3 and Supplementary Table 1-2.

Supplementary Figures are in PowerPoints File.

Supplementary Tables are in Word File.
