# Supplementary material for: Prognostic impact of systolic blood pressure and antithrombotic strategy in patients with atrial fibrillation and stable coronary artery disease: a post-hoc analysis of the AFIRE trial
Source: Hypertens Res. 2026 Jan 5;49(4):1139–49. doi: 10.1038/s41440-025-02449-9 (PMC13050638; doi:10.1038/s41440-025-02449-9)
Supplement: Supplementary file 5 — The list of AFIRE investigators [file 41440_2025_2449_MOESM5_ESM.docx]

**The list of AFIRE investigators**

[Akihiro Nakamura](https://pubmed.ncbi.nlm.nih.gov/?term=Nakamura+A), [Eiji Tamiya](https://pubmed.ncbi.nlm.nih.gov/?term=Tamiya+E), [Tadashi Yamamoto](https://pubmed.ncbi.nlm.nih.gov/?term=Yamamoto+T), [Syuji Suetake](https://pubmed.ncbi.nlm.nih.gov/?term=Suetake+S), [Teruo Noguchi](https://pubmed.ncbi.nlm.nih.gov/?term=Noguchi+T), [Shinichi Nakamura](https://pubmed.ncbi.nlm.nih.gov/?term=Nakamura+S), [Akihiko Matsumura](https://pubmed.ncbi.nlm.nih.gov/?term=Matsumura+A), [Jisho Kojima](https://pubmed.ncbi.nlm.nih.gov/?term=Kojima+J), [Hiroshi Yamaguchi](https://pubmed.ncbi.nlm.nih.gov/?term=Yamaguchi+H), [Satoru Suwa](https://pubmed.ncbi.nlm.nih.gov/?term=Suwa+S), [Takanori Yasu](https://pubmed.ncbi.nlm.nih.gov/?term=Yasu+T), [Akihiro Nakajima](https://pubmed.ncbi.nlm.nih.gov/?term=Nakajima+A), [Takahisa Yamada](https://pubmed.ncbi.nlm.nih.gov/?term=Yamada+T), [Hidekazu Arai](https://pubmed.ncbi.nlm.nih.gov/?term=Arai+H), [Yoshiki Hata](https://pubmed.ncbi.nlm.nih.gov/?term=Hata+Y), [Toshihiko Sakanashi](https://pubmed.ncbi.nlm.nih.gov/?term=Sakanashi+T), [Hironobu Tateishi](https://pubmed.ncbi.nlm.nih.gov/?term=Tateishi+H), [Toru Nakayama](https://pubmed.ncbi.nlm.nih.gov/?term=Nakayama+T), [Yoichi Nozaki](https://pubmed.ncbi.nlm.nih.gov/?term=Nozaki+Y), [Yasuo Okumura](https://pubmed.ncbi.nlm.nih.gov/?term=Okumura+Y), [Masahide Tokue](https://pubmed.ncbi.nlm.nih.gov/?term=Tokue+M), [Norihiro Kuroki](https://pubmed.ncbi.nlm.nih.gov/?term=Kuroki+N), [Yasuyuki Maruyama](https://pubmed.ncbi.nlm.nih.gov/?term=Maruyama+Y), [Hiroshi Suzuki](https://pubmed.ncbi.nlm.nih.gov/?term=Suzuki+H), [Yasunori Nishida](https://pubmed.ncbi.nlm.nih.gov/?term=Nishida+Y), [Masazumi Ajioka](https://pubmed.ncbi.nlm.nih.gov/?term=Ajioka+M), [Kazuhiko Yumoto](https://pubmed.ncbi.nlm.nih.gov/?term=Yumoto+K), [Shogo Shimizu](https://pubmed.ncbi.nlm.nih.gov/?term=Shimizu+S), [Takahiko Aoyama](https://pubmed.ncbi.nlm.nih.gov/?term=Aoyama+T), [Hideki Shimomura](https://pubmed.ncbi.nlm.nih.gov/?term=Shimomura+H), [Terunori Takeda](https://pubmed.ncbi.nlm.nih.gov/?term=Takeda+T), [Koichi Oshiro](https://pubmed.ncbi.nlm.nih.gov/?term=Oshiro+K), [Nobuyoshi Sugishita](https://pubmed.ncbi.nlm.nih.gov/?term=Sugishita+N), [Yoshisato Shibata](https://pubmed.ncbi.nlm.nih.gov/?term=Shibata+Y), [Takatoshi Otonari](https://pubmed.ncbi.nlm.nih.gov/?term=Otonari+T), [Makoto Shimizu](https://pubmed.ncbi.nlm.nih.gov/?term=Shimizu+M), [Hajime Kihara](https://pubmed.ncbi.nlm.nih.gov/?term=Kihara+H), [Hiroshi Ogawa](https://pubmed.ncbi.nlm.nih.gov/?term=Ogawa+H), [Atsuyuki Ono](https://pubmed.ncbi.nlm.nih.gov/?term=Ono+A), [Minoru Hazama](https://pubmed.ncbi.nlm.nih.gov/?term=Hazama+M), [Kengo Tsukahara](https://pubmed.ncbi.nlm.nih.gov/?term=Tsukahara+K), [Seichi Haruta](https://pubmed.ncbi.nlm.nih.gov/?term=Haruta+S), [Tetsuya Haruna](https://pubmed.ncbi.nlm.nih.gov/?term=Haruna+T), [Masaaki Ito](https://pubmed.ncbi.nlm.nih.gov/?term=Ito+M), [Kenji Fujii](https://pubmed.ncbi.nlm.nih.gov/?term=Fujii+K), [Nobuhiko Atsuchi](https://pubmed.ncbi.nlm.nih.gov/?term=Atsuchi+N), [Masataka Sata](https://pubmed.ncbi.nlm.nih.gov/?term=Sata+M), [Takatoshi Wakeyama](https://pubmed.ncbi.nlm.nih.gov/?term=Wakeyama+T), [Naoyuki Hasebe](https://pubmed.ncbi.nlm.nih.gov/?term=Hasebe+N), [Yoshio Kobayasi](https://pubmed.ncbi.nlm.nih.gov/?term=Kobayasi+Y), [Kazuo Osato](https://pubmed.ncbi.nlm.nih.gov/?term=Osato+K), [Kiyoshi Hironaga](https://pubmed.ncbi.nlm.nih.gov/?term=Hironaga+K), [Yujiro Naganuma](https://pubmed.ncbi.nlm.nih.gov/?term=Naganuma+Y), [Kazuhiro Anzaki](https://pubmed.ncbi.nlm.nih.gov/?term=Anzaki+K), [Shinya Okazaki](https://pubmed.ncbi.nlm.nih.gov/?term=Okazaki+S), [Yusuke Nakagawa](https://pubmed.ncbi.nlm.nih.gov/?term=Nakagawa+Y), [Keichi Tokuhiro](https://pubmed.ncbi.nlm.nih.gov/?term=Tokuhiro+K), [Koichi Tanaka](https://pubmed.ncbi.nlm.nih.gov/?term=Tanaka+K), [Tomoyasu Momose](https://pubmed.ncbi.nlm.nih.gov/?term=Momose+T), [Yusuke Fukushima](https://pubmed.ncbi.nlm.nih.gov/?term=Fukushima+Y), [Ryosuke Kametani](https://pubmed.ncbi.nlm.nih.gov/?term=Kametani+R), [Katsunori Kawamitsu](https://pubmed.ncbi.nlm.nih.gov/?term=Kawamitsu+K), [Yoshihiko Saito](https://pubmed.ncbi.nlm.nih.gov/?term=Saito+Y), [Shintaro Akashi](https://pubmed.ncbi.nlm.nih.gov/?term=Akashi+S), [Kenta Kumagai](https://pubmed.ncbi.nlm.nih.gov/?term=Kumagai+K), [Kenichi Eshima](https://pubmed.ncbi.nlm.nih.gov/?term=Eshima+K), [Tetsuya Tobaru](https://pubmed.ncbi.nlm.nih.gov/?term=Tobaru+T), [Toshihiko Seo](https://pubmed.ncbi.nlm.nih.gov/?term=Seo+T), [Koichiro Okuhara](https://pubmed.ncbi.nlm.nih.gov/?term=Okuhara+K), [Ken Kozuma](https://pubmed.ncbi.nlm.nih.gov/?term=Kozuma+K), [Yuji Ikari](https://pubmed.ncbi.nlm.nih.gov/?term=Ikari+Y), [Toshiyuki Takahashi](https://pubmed.ncbi.nlm.nih.gov/?term=Takahashi+T), [Koji Oiwa](https://pubmed.ncbi.nlm.nih.gov/?term=Oiwa+K), [Ichiro Michishita](https://pubmed.ncbi.nlm.nih.gov/?term=Michishita+I), [Hisanori Fujikura](https://pubmed.ncbi.nlm.nih.gov/?term=Fujikura+H), [Shinichi Momomura](https://pubmed.ncbi.nlm.nih.gov/?term=Momomura+S), [Yoshihiro Yamamoto](https://pubmed.ncbi.nlm.nih.gov/?term=Yamamoto+Y), [Kenichiro Otomo](https://pubmed.ncbi.nlm.nih.gov/?term=Otomo+K), [Taku Matsubara](https://pubmed.ncbi.nlm.nih.gov/?term=Matsubara+T), [Hideki Tashiro](https://pubmed.ncbi.nlm.nih.gov/?term=Tashiro+H), [Teruo Inoue](https://pubmed.ncbi.nlm.nih.gov/?term=Inoue+T), [Masaharu Ishihara](https://pubmed.ncbi.nlm.nih.gov/?term=Ishihara+M), [Ichiro Shiojima](https://pubmed.ncbi.nlm.nih.gov/?term=Shiojima+I), [Eizo Tachibana](https://pubmed.ncbi.nlm.nih.gov/?term=Tachibana+E), [Kotaro Sumii](https://pubmed.ncbi.nlm.nih.gov/?term=Sumii+K), [Nobuyasu Yamamoto](https://pubmed.ncbi.nlm.nih.gov/?term=Yamamoto+N), [Nobuhiro Omura](https://pubmed.ncbi.nlm.nih.gov/?term=Omura+N), [Toshihiro Nakamura](https://pubmed.ncbi.nlm.nih.gov/?term=Nakamura+T), [Naohiko Takahashi](https://pubmed.ncbi.nlm.nih.gov/?term=Takahashi+N), [Yukiko Morita](https://pubmed.ncbi.nlm.nih.gov/?term=Morita+Y), [Kouki Watanabe](https://pubmed.ncbi.nlm.nih.gov/?term=Watanabe+K), [Hiroyuki Fujinaga](https://pubmed.ncbi.nlm.nih.gov/?term=Fujinaga+H), [Michiro Maruyama](https://pubmed.ncbi.nlm.nih.gov/?term=Maruyama+M), [Takefumi Oka](https://pubmed.ncbi.nlm.nih.gov/?term=Oka+T), [Takeshi Shirayama](https://pubmed.ncbi.nlm.nih.gov/?term=Shirayama+T), [Tetsuya Amano](https://pubmed.ncbi.nlm.nih.gov/?term=Amano+T), [Kazuki Fukui](https://pubmed.ncbi.nlm.nih.gov/?term=Fukui+K), [Kenji Ando](https://pubmed.ncbi.nlm.nih.gov/?term=Ando+K), [Shuichi Oshima](https://pubmed.ncbi.nlm.nih.gov/?term=Oshima+S), [Shuntaro Kagiyama](https://pubmed.ncbi.nlm.nih.gov/?term=Kagiyama+S), [Hiroki Teragawa](https://pubmed.ncbi.nlm.nih.gov/?term=Teragawa+H), [Masaru Yuge](https://pubmed.ncbi.nlm.nih.gov/?term=Yuge+M), [Shiro Ono](https://pubmed.ncbi.nlm.nih.gov/?term=Ono+S), [Tokushi Koga](https://pubmed.ncbi.nlm.nih.gov/?term=Koga+T), [Katsuhito Fujiu](https://pubmed.ncbi.nlm.nih.gov/?term=Fujiu+K), [Masanari Kuwabara](https://pubmed.ncbi.nlm.nih.gov/?term=Kuwabara+M), [Yusuke Oya](https://pubmed.ncbi.nlm.nih.gov/?term=Oya+Y), [Yoshihiro Yumoto](https://pubmed.ncbi.nlm.nih.gov/?term=Yumoto+Y), [Naomitsu Kuji](https://pubmed.ncbi.nlm.nih.gov/?term=Kuji+N), [Makoto Ikemura](https://pubmed.ncbi.nlm.nih.gov/?term=Ikemura+M), [Kazuomi Kario](https://pubmed.ncbi.nlm.nih.gov/?term=Kario+K), [Kenichi Chatani](https://pubmed.ncbi.nlm.nih.gov/?term=Chatani+K), [Koji Sato](https://pubmed.ncbi.nlm.nih.gov/?term=Sato+K), [Hiroo Miyagi](https://pubmed.ncbi.nlm.nih.gov/?term=Miyagi+H), [Mikitaka Murakami](https://pubmed.ncbi.nlm.nih.gov/?term=Murakami+M), [Katumi Saito](https://pubmed.ncbi.nlm.nih.gov/?term=Saito+K), [Masaaki Hoshiga](https://pubmed.ncbi.nlm.nih.gov/?term=Hoshiga+M), [Shinji Sato](https://pubmed.ncbi.nlm.nih.gov/?term=Sato+S), [Norifumi Kubo](https://pubmed.ncbi.nlm.nih.gov/?term=Kubo+N), [Yasuhiro Sakamoto](https://pubmed.ncbi.nlm.nih.gov/?term=Sakamoto+Y), [Kazuhiro Ashida](https://pubmed.ncbi.nlm.nih.gov/?term=Ashida+K), [Hiroki Sakamoto](https://pubmed.ncbi.nlm.nih.gov/?term=Sakamoto+H), [Saroshi Murasaki](https://pubmed.ncbi.nlm.nih.gov/?term=Murasaki+S), [Hiroki Uehara](https://pubmed.ncbi.nlm.nih.gov/?term=Uehara+H), [Takashi Akasaka](https://pubmed.ncbi.nlm.nih.gov/?term=Akasaka+T), [Yurika Oba](https://pubmed.ncbi.nlm.nih.gov/?term=Oba+Y), [Shiro Nakahara](https://pubmed.ncbi.nlm.nih.gov/?term=Nakahara+S), [Yoichi Hanaoka](https://pubmed.ncbi.nlm.nih.gov/?term=Hanaoka+Y), [Toshitaka Nishimiya](https://pubmed.ncbi.nlm.nih.gov/?term=Nishimiya+T), [Ryusuke Tsunoda](https://pubmed.ncbi.nlm.nih.gov/?term=Tsunoda+R), [Yoshito Onuma](https://pubmed.ncbi.nlm.nih.gov/?term=Onuma+Y), [Sadanobu Higuchi](https://pubmed.ncbi.nlm.nih.gov/?term=Higuchi+S), [Akihiro Tani](https://pubmed.ncbi.nlm.nih.gov/?term=Tani+A), [Atsuyuki Wada](https://pubmed.ncbi.nlm.nih.gov/?term=Wada+A), [Masashi Kato](https://pubmed.ncbi.nlm.nih.gov/?term=Kato+M), [Hiromi Obata](https://pubmed.ncbi.nlm.nih.gov/?term=Obata+H), [Yoshiharu Higuchi](https://pubmed.ncbi.nlm.nih.gov/?term=Higuchi+Y), [Tsutomu Endo](https://pubmed.ncbi.nlm.nih.gov/?term=Endo+T), [Ritsushi Kato](https://pubmed.ncbi.nlm.nih.gov/?term=Kato+R), [Toshiro Matsunaga](https://pubmed.ncbi.nlm.nih.gov/?term=Matsunaga+T), [Tosizo Matsuoka](https://pubmed.ncbi.nlm.nih.gov/?term=Matsuoka+T), [Hiroo Noguchi](https://pubmed.ncbi.nlm.nih.gov/?term=Noguchi+H), [Makoto Usui](https://pubmed.ncbi.nlm.nih.gov/?term=Usui+M), [Takahiro Hayashi](https://pubmed.ncbi.nlm.nih.gov/?term=Hayashi+T), [Yutaka Otsuji](https://pubmed.ncbi.nlm.nih.gov/?term=Otsuji+Y), [Takuya Osaki](https://pubmed.ncbi.nlm.nih.gov/?term=Osaki+T), [Hirofumi Zaizen](https://pubmed.ncbi.nlm.nih.gov/?term=Zaizen+H), [Hirotaka Yoshihara](https://pubmed.ncbi.nlm.nih.gov/?term=Yoshihara+H), [Kazushige Kadota](https://pubmed.ncbi.nlm.nih.gov/?term=Kadota+K), [Toyoki Hirose](https://pubmed.ncbi.nlm.nih.gov/?term=Hirose+T), [Takuya Miyazawa](https://pubmed.ncbi.nlm.nih.gov/?term=Miyazawa+T), [Atsushi Mori](https://pubmed.ncbi.nlm.nih.gov/?term=Mori+A), [Masamitsu Takano](https://pubmed.ncbi.nlm.nih.gov/?term=Takano+M), [Wataru Shimizu](https://pubmed.ncbi.nlm.nih.gov/?term=Shimizu+W), [Minoru Wake](https://pubmed.ncbi.nlm.nih.gov/?term=Wake+M), [Seizo Oriso](https://pubmed.ncbi.nlm.nih.gov/?term=Oriso+S), [Minoru Yoshiyama](https://pubmed.ncbi.nlm.nih.gov/?term=Yoshiyama+M), [Shigeo Kakinoki](https://pubmed.ncbi.nlm.nih.gov/?term=Kakinoki+S), [Toshihiko Nishioka](https://pubmed.ncbi.nlm.nih.gov/?term=Nishioka+T), [Takefumi Ozaki](https://pubmed.ncbi.nlm.nih.gov/?term=Ozaki+T), [Kazumiki Nomoto](https://pubmed.ncbi.nlm.nih.gov/?term=Nomoto+K), [Kosaburo Seki](https://pubmed.ncbi.nlm.nih.gov/?term=Seki+K), [Kazuya Kawai](https://pubmed.ncbi.nlm.nih.gov/?term=Kawai+K), [Yukio Ozaki](https://pubmed.ncbi.nlm.nih.gov/?term=Ozaki+Y), [Shinichiro Miura](https://pubmed.ncbi.nlm.nih.gov/?term=Miura+S), [Masanori Kawasaki](https://pubmed.ncbi.nlm.nih.gov/?term=Kawasaki+M), [Ryuichi Funada](https://pubmed.ncbi.nlm.nih.gov/?term=Funada+R), [Keigo Dote](https://pubmed.ncbi.nlm.nih.gov/?term=Dote+K), [Shinya Okamoto](https://pubmed.ncbi.nlm.nih.gov/?term=Okamoto+S), [Takayuki Owada](https://pubmed.ncbi.nlm.nih.gov/?term=Owada+T), [Tomohiro Doke](https://pubmed.ncbi.nlm.nih.gov/?term=Doke+T), [Toshiyuki Matsumura](https://pubmed.ncbi.nlm.nih.gov/?term=Matsumura+T), [Toshihiko Kubo](https://pubmed.ncbi.nlm.nih.gov/?term=Kubo+T), [Masataka Horiuchi](https://pubmed.ncbi.nlm.nih.gov/?term=Horiuchi+M), [Toshihiko Nagano](https://pubmed.ncbi.nlm.nih.gov/?term=Nagano+T), [Atsushi Takaishi](https://pubmed.ncbi.nlm.nih.gov/?term=Takaishi+A), [Masaya Yamamoto](https://pubmed.ncbi.nlm.nih.gov/?term=Yamamoto+M), [Hitoshi Nakashima](https://pubmed.ncbi.nlm.nih.gov/?term=Nakashima+H), [Yukichi Murozono](https://pubmed.ncbi.nlm.nih.gov/?term=Murozono+Y), [Mitsuru Munemasa](https://pubmed.ncbi.nlm.nih.gov/?term=Munemasa+M), [Yasushi Sakata](https://pubmed.ncbi.nlm.nih.gov/?term=Sakata+Y), [Naoto Inoue](https://pubmed.ncbi.nlm.nih.gov/?term=Inoue+N), [Takehiro Ota](https://pubmed.ncbi.nlm.nih.gov/?term=Ota+T), [Yutaka Hamano](https://pubmed.ncbi.nlm.nih.gov/?term=Hamano+Y), [Norihiro Abe](https://pubmed.ncbi.nlm.nih.gov/?term=Abe+N), [Toshio Tsubokura](https://pubmed.ncbi.nlm.nih.gov/?term=Tsubokura+T), [Masami Goto](https://pubmed.ncbi.nlm.nih.gov/?term=Goto+M), [Isao Kubota](https://pubmed.ncbi.nlm.nih.gov/?term=Kubota+I), [Masafumi Yano](https://pubmed.ncbi.nlm.nih.gov/?term=Yano+M), [Ken Umetani](https://pubmed.ncbi.nlm.nih.gov/?term=Umetani+K), [Taro Date](https://pubmed.ncbi.nlm.nih.gov/?term=Date+T), [Hideo Morimoto](https://pubmed.ncbi.nlm.nih.gov/?term=Morimoto+H), [Toshiyuki Noda](https://pubmed.ncbi.nlm.nih.gov/?term=Noda+T), [Seiichi Goto](https://pubmed.ncbi.nlm.nih.gov/?term=Goto+S), [Kiyoshi Hibi](https://pubmed.ncbi.nlm.nih.gov/?term=Hibi+K), [Akira Nakano](https://pubmed.ncbi.nlm.nih.gov/?term=Nakano+A), [Shinya Hiramitsu](https://pubmed.ncbi.nlm.nih.gov/?term=Hiramitsu+S), [Yasuki Kihara](https://pubmed.ncbi.nlm.nih.gov/?term=Kihara+Y), [Masafumi Sugi](https://pubmed.ncbi.nlm.nih.gov/?term=Sugi+M), [Nobuyuki Shiba](https://pubmed.ncbi.nlm.nih.gov/?term=Shiba+N), [Daisuke Izumi](https://pubmed.ncbi.nlm.nih.gov/?term=Izumi+D), [Tetsuya Sato](https://pubmed.ncbi.nlm.nih.gov/?term=Sato+T), [Kosuke Ajiki](https://pubmed.ncbi.nlm.nih.gov/?term=Ajiki+K), [Mitsuru Oishi](https://pubmed.ncbi.nlm.nih.gov/?term=Oishi+M), [Michiro Kiryu](https://pubmed.ncbi.nlm.nih.gov/?term=Kiryu+M), [Tenei Ko](https://pubmed.ncbi.nlm.nih.gov/?term=Ko+T), [Hideyuki Ando](https://pubmed.ncbi.nlm.nih.gov/?term=Ando+H), [Syunichi Miyazaki](https://pubmed.ncbi.nlm.nih.gov/?term=Miyazaki+S), [Toru Kinugawa](https://pubmed.ncbi.nlm.nih.gov/?term=Kinugawa+T), [Hiromasa Otake](https://pubmed.ncbi.nlm.nih.gov/?term=Otake+H), [Hiroaki Kitaoka](https://pubmed.ncbi.nlm.nih.gov/?term=Kitaoka+H), [Shinji Tayama](https://pubmed.ncbi.nlm.nih.gov/?term=Tayama+S), [Yoshihiro Hirata](https://pubmed.ncbi.nlm.nih.gov/?term=Hirata+Y), [Soichi Honda](https://pubmed.ncbi.nlm.nih.gov/?term=Honda+S), [Mamoru Manita](https://pubmed.ncbi.nlm.nih.gov/?term=Manita+M), [Yasuhiro Ishii](https://pubmed.ncbi.nlm.nih.gov/?term=Ishii+Y), [Hiroyuki Oka](https://pubmed.ncbi.nlm.nih.gov/?term=Oka+H), [Yasuharu Nanba](https://pubmed.ncbi.nlm.nih.gov/?term=Nanba+Y), [Masami Nishino](https://pubmed.ncbi.nlm.nih.gov/?term=Nishino+M), [Tomohiro Sakamoto](https://pubmed.ncbi.nlm.nih.gov/?term=Sakamoto+T), [Toshihiko Saito](https://pubmed.ncbi.nlm.nih.gov/?term=Saito+T), [Hirosumi Sakai](https://pubmed.ncbi.nlm.nih.gov/?term=Sakai+H), [Makoto Ichikawa](https://pubmed.ncbi.nlm.nih.gov/?term=Ichikawa+M), [Shigeto Namiuchi](https://pubmed.ncbi.nlm.nih.gov/?term=Namiuchi+S), [Toshiki Matsui](https://pubmed.ncbi.nlm.nih.gov/?term=Matsui+T), [Kanichi Inoue](https://pubmed.ncbi.nlm.nih.gov/?term=Inoue+K), [Nobuyuki Komiyama](https://pubmed.ncbi.nlm.nih.gov/?term=Komiyama+N), [Yoshihiro Akashi](https://pubmed.ncbi.nlm.nih.gov/?term=Akashi+Y), [Yuichi Nakamura](https://pubmed.ncbi.nlm.nih.gov/?term=Nakamura+Y), [Tatsuya Komaru](https://pubmed.ncbi.nlm.nih.gov/?term=Komaru+T), [Takeshi Hosokawa](https://pubmed.ncbi.nlm.nih.gov/?term=Hosokawa+T), [Taishiro Chikamori](https://pubmed.ncbi.nlm.nih.gov/?term=Chikamori+T), [Hiroyuki Tanaka](https://pubmed.ncbi.nlm.nih.gov/?term=Tanaka+H), [Atsushi Suzuki](https://pubmed.ncbi.nlm.nih.gov/?term=Suzuki+A), [Osamu Arasaki](https://pubmed.ncbi.nlm.nih.gov/?term=Arasaki+O), [Kazutaka Aonuma](https://pubmed.ncbi.nlm.nih.gov/?term=Aonuma+K), [Yutaka Wakasa](https://pubmed.ncbi.nlm.nih.gov/?term=Wakasa+Y), [Tomoharu Yoshizawa](https://pubmed.ncbi.nlm.nih.gov/?term=Yoshizawa+T), [Teruyasu Sugano](https://pubmed.ncbi.nlm.nih.gov/?term=Sugano+T), [Naoto Yokota](https://pubmed.ncbi.nlm.nih.gov/?term=Yokota+N), [Akiyoshi Kakutani](https://pubmed.ncbi.nlm.nih.gov/?term=Kakutani+A), [Tomohiro Suzuki](https://pubmed.ncbi.nlm.nih.gov/?term=Suzuki+T), [Yusei Abe](https://pubmed.ncbi.nlm.nih.gov/?term=Abe+Y), [Toru Kataoka](https://pubmed.ncbi.nlm.nih.gov/?term=Kataoka+T), [Hideki Okayama](https://pubmed.ncbi.nlm.nih.gov/?term=Okayama+H), [Hiroyoshi Yokoi](https://pubmed.ncbi.nlm.nih.gov/?term=Yokoi+H), [Keiichi Chin](https://pubmed.ncbi.nlm.nih.gov/?term=Chin+K), [Koichi Hasegawa](https://pubmed.ncbi.nlm.nih.gov/?term=Hasegawa+K), [Hirofumi Tomita](https://pubmed.ncbi.nlm.nih.gov/?term=Tomita+H), [Hirotsugu Honzyo](https://pubmed.ncbi.nlm.nih.gov/?term=Honzyo+H), [Hiroya Kawai](https://pubmed.ncbi.nlm.nih.gov/?term=Kawai+H), [Kazuya Yamamoto](https://pubmed.ncbi.nlm.nih.gov/?term=Yamamoto+K), [Yoshihiro Morino](https://pubmed.ncbi.nlm.nih.gov/?term=Morino+Y), [Shuji Tsujiyama](https://pubmed.ncbi.nlm.nih.gov/?term=Tsujiyama+S), [Michihiro Yoshimura](https://pubmed.ncbi.nlm.nih.gov/?term=Yoshimura+M), [Syuichi Hamasaki](https://pubmed.ncbi.nlm.nih.gov/?term=Hamasaki+S), [Yawara Niijima](https://pubmed.ncbi.nlm.nih.gov/?term=Niijima+Y), [Takuma Aoyama](https://pubmed.ncbi.nlm.nih.gov/?term=Aoyama+T), [Yuji Mizuno](https://pubmed.ncbi.nlm.nih.gov/?term=Mizuno+Y), [Akira Maki](https://pubmed.ncbi.nlm.nih.gov/?term=Maki+A), [Kengo Tanabe](https://pubmed.ncbi.nlm.nih.gov/?term=Tanabe+K), [Toyoaki Murohara](https://pubmed.ncbi.nlm.nih.gov/?term=Murohara+T), [Tomoki Nakamura](https://pubmed.ncbi.nlm.nih.gov/?term=Nakamura+T), [Shojiro Naomi](https://pubmed.ncbi.nlm.nih.gov/?term=Naomi+S), [Naoya Matsumoto](https://pubmed.ncbi.nlm.nih.gov/?term=Matsumoto+N), [Tohru Minamino](https://pubmed.ncbi.nlm.nih.gov/?term=Minamino+T), [Hiroshige Sairenji](https://pubmed.ncbi.nlm.nih.gov/?term=Sairenji+H), [Noriyuki Miyamoto](https://pubmed.ncbi.nlm.nih.gov/?term=Miyamoto+N), [Masaya Arikawa](https://pubmed.ncbi.nlm.nih.gov/?term=Arikawa+M), [Hiroshi Ito](https://pubmed.ncbi.nlm.nih.gov/?term=Ito+H), [Yoshifusa Matsuura](https://pubmed.ncbi.nlm.nih.gov/?term=Matsuura+Y), [Shiro Hata](https://pubmed.ncbi.nlm.nih.gov/?term=Hata+S), [Yusuke Nakatsu](https://pubmed.ncbi.nlm.nih.gov/?term=Nakatsu+Y), [Tomoya Onodera](https://pubmed.ncbi.nlm.nih.gov/?term=Onodera+T), [Toru Kato](https://pubmed.ncbi.nlm.nih.gov/?term=Kato+T), [Hideo Amano](https://pubmed.ncbi.nlm.nih.gov/?term=Amano+H), [Eiichi Tokutake](https://pubmed.ncbi.nlm.nih.gov/?term=Tokutake+E), [Masafumi Kasao](https://pubmed.ncbi.nlm.nih.gov/?term=Kasao+M), [Masahiko Moriguchi](https://pubmed.ncbi.nlm.nih.gov/?term=Moriguchi+M), [Kazuhiro Yamamoto](https://pubmed.ncbi.nlm.nih.gov/?term=Yamamoto+K), [Masazumi Tsuji](https://pubmed.ncbi.nlm.nih.gov/?term=Tsuji+M), [Hareaki Yamamoto](https://pubmed.ncbi.nlm.nih.gov/?term=Yamamoto+H), [Yuzuru Yanbe](https://pubmed.ncbi.nlm.nih.gov/?term=Yanbe+Y), [Takamasa Iwasawa](https://pubmed.ncbi.nlm.nih.gov/?term=Iwasawa+T), [Makoto Suzuki](https://pubmed.ncbi.nlm.nih.gov/?term=Suzuki+M), [Hisao Mori](https://pubmed.ncbi.nlm.nih.gov/?term=Mori+H), [Eiji Shibahashi](https://pubmed.ncbi.nlm.nih.gov/?term=Shibahashi+E), [Masahiro Takita](https://pubmed.ncbi.nlm.nih.gov/?term=Takita+M), [Kazuo Kimura](https://pubmed.ncbi.nlm.nih.gov/?term=Kimura+K)
